# Elastic Wave Application for Damage Detection in Concrete Slab with GFRP Reinforcement

**DOI:** 10.3390/ma15238523

**Published:** 2022-11-29

**Authors:** Dominika Ziaja, Michał Jurek, Agnieszka Wiater

**Affiliations:** 1 Department of Structural Mechanics, Rzeszow University of Technology, ul. Poznańska 2, 35-084 Rzeszów, Poland; 2 Department of Roads and Bridges, Rzeszow University of Technology, ul. Poznańska 2, 35-084 Rzeszów, Poland

**Keywords:** elastic waves, digital image correlation, concrete slabs, GFRP reinforcement, cracks

## Abstract

The aim of the presented examination is condition-monitoring of GFRP-reinforced concrete structural members using elastic wave propagation. As an example, a deck slab is selected. The deck slab is made of concrete of the targeted C30/37 class under three-point bending. During loading cycles, the specimen is observed with a digital image correlation (DIC) system, which enables calculation of the strain field. The measuring setup consists of two Baumer 12.3 Mpx cameras with VS-1220HV lenses, combined in a Q400 system by Dantec Dynamics GmbH. Elastic waves are also measured based on signals recorded with PZT (lead–zirconate–titanate) sensors. Additionally, the typical crack-opening measurements are made. The appearance of a crack and its growth causes changes in both the shape and amplitude of the registered signals. However, the changes are not obvious and depend on the location of the sensors. Due to the impossibility of determining simple parameters with respect to disturbingly wide cracks, for damage detection, an artificial neural network (ANN) is applied. Perfect determination of the specimen’s condition (100% properly classified patterns) is possible based on whether the element is under loading or not.

## 1. Introduction

Concrete, as a very popular construction material, is widely applied in many structural members and works in varying loading conditions. Due to its properties, this material transfers compression much better than tension, so in elements with tensile zones (but not exclusively), the application of reinforcement is necessary. Additionally, the inhomogeneous internal structure of this material makes the exact prediction of crack appearance and its propagation path impossible. Despite the use of reinforcement in concrete structures, cracks appear; however, they do not always mean that the operation of the facility becomes unsafe. A certain acceptable level of cracks is assumed, which allows continued safe use of the structure.

Control of cracking in reinforced concrete is important due to aesthetic reasons and to prevent water leakage. Other aspects considered in crack limitation are creep cracking and shear effects. The load-bearing elements of a structure in which cracks have appeared should be regularly observed and examined in detail. The presence of cracks with large width and height may be a signal of deterioration of the structure’s condition, leading to its uselessness.

Crack-opening measurements are most often performed manually using a feeler or fracture-width measuring instrument during periodic or ad hoc inspections. Like any manual control, it requires involvement of an experienced and skilled operator and gives a view of the structure only during the inspection. Until the next inspection, the situation may change as a result of exploitation. Not surprisingly, more and more scientists are looking for alternative ways to detect and assess cracks in concrete elements, especially in regards to construction that would have high consequences if damaged [1].

Among many non-destructive testing (NDT) techniques in crack detection, the following are applied: elastic wave propagation measurements [2,3], ultrasonic coda wave interferometry [4], digital image correlation [5], X-ray tomography [6], acoustic emission [1], and impedance-based methods [7,8]. Two of these methods are used in this examination, namely elastic wave propagation and DIC.

Elastic wave propagation through a material is very similar to how a wave propagates in water. Waves can propagate inside the material [9] or along its surface [10]. When they encounter an obstacle, they reflect on it, changing the character of propagation. The observation of wave propagation enables the detection of changes in material and/or structural elements. The most popular method of elastic wave measurement in concrete structures is contact measurement with PZT transducers, which can be embedded inside the examined element [2,11] or glued onto its surface [10,12].

Elastic waves are applied not only for crack detection in concrete structural members but also in different types of materials, such as metal [13] or composite [14], or in damage detection of connections [15]. This phenomenon is also used for the detection of reinforcement bar anchorage length [16] and in many other applications.

In concrete specimens, where the exact location of failure and the way of its propagation are difficult to predict, simultaneous measurements at many points is highly desirable. This opportunity is given by DIC [17]. The main idea of this measurement method is a comparison of the sequence of photos registered with calibrated cameras. Changes in pictures, after taking into account the calibration data, can be correlated to changes in observed areas (in terms of displacements and deformations). Thanks to this, this method enables obtaining information about strain/displacement fields in the whole observed region without the intervention of mass or surface properties of the examined element [18].

Examples of DIC application can be found: [4] for crack propagation observation in concrete cube specimens under wedge splitting test; [5] for crack mouth opening measurements in reinforced concrete beams under three-point bending; [19] for 3D displacement measurements in reinforced concrete beams under torsion; and [20] for fatigue crack monitoring in mild steel specimens. It is worth mentioning that other digital image processing methods are also being tried in crack measurement [21]; some of them do not require special equipment [22]—a smartphone is enough.

The purpose of this study is to determine the usefulness of the propagation of an elastic wave to determine the alarm level of crack opening. It is the continuation of research presented in [23] involving real-sized structural elements, but this time, we use a deck slab as a sample and the examination is enriched with DIC measurements. Thanks to implementation of DIC in this research, the crack growth history can be observed and the influence of crack depth on wave propagation can be considered.

In the literature, a few tasks similar to those presented in this paper can be found. Kee and Nam [12] examined the feasibility of the application of PZT disk sensors mounted on a beam’s surface to generate and receive the elastic wave in the crack detection problem. They showed that the surface wave is sensitive to cracks that are deeper than about 80 % of the wavelength. However, their choices for type, location, and distance between the sensors are significantly different than those in our examination.

Localization of the cracks in concrete based on signals collected with PZT sensors is also the aim of the study by Narayanan et al. [10]. Likewise, they used DIC for crack penetration measurements, but the size of the sample they used was smaller (500 mm × 150 mm × 150 mm) and four-point bending was applied. Additionally, they analyzed the crack’s influence on the impedance of the system. They stated that electromagnetic impedance, as well as stress-wave propagation techniques, are sensitive to discontinuities produced by cracks in the concrete.

The same-sized small (500 mm × 150 mm × 150 mm) concrete specimen but under a three-point bending test was an object of examination by Kocherla et al. [2]. The authors used embedded PZT sensors for monitoring crack formation and opening. The process was also observed with DIC. In the conclusion, they stated that the presence of a discontinuity in the material results in a decrease in signal amplitude. The time of wave flight increases, too.

In the above-mentioned papers, damage indexes or factors that are sensitive to cracks are used. In this publication, artificial intelligence is used to determine whether the slab can be still exploited or not. Shallow artificial neural networks, which have been previously trained, classify the specimen’s condition. ANNs have often been applied in this type of task, enabling automation of the damage-detection process [24,25,26,27,28,29]. For a properly trained tool, the presence of a specialist during results analysis is not necessary.

Another interesting application of elastic waves to damage-imaging in concrete slabs was presented in [30]. The authors used six embedded piezoceramic transducers to detect a hole in a 900 mm × 900 mm × 20 mm slab. Despite their presented results being promising, it should be noted that the specimen was not reinforced nor loaded. Thus, application of this method to real engineering structures requires consideration of many more factors.

It can be mentioned that not only elastic waves are used for NDT of concrete slabs. In the literature, examples of the application of vibration-based methods are also presented, such as in [31]. However, it is difficult to find the possibility of the application of single-slab condition monitoring based on its natural vibration if the slab is embedded into a bigger structure such as a bridge. This method is useful rather in laboratory tests.

## 2. Materials and Methods

**Figure 1:**
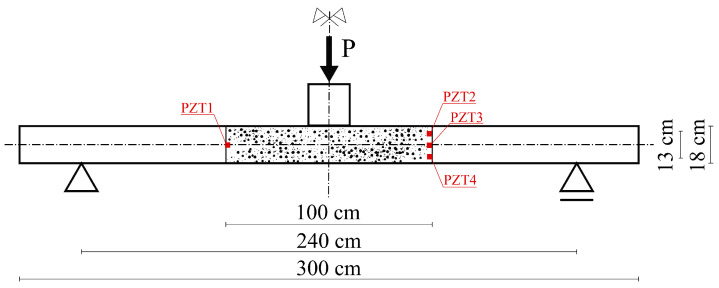
Dimensions of the specimen, location of observed area, and scheme of sensor locations.

The prepared experiment relied on the observation of part of a deck-slab side surface under the three-point bending test. The deck slab width was 100 cm, the other dimensions of the slab as well as the location of the observed area are shown in Figure 1. The dimensions of the slab specimen correspond to the typical dimensions of a road bridge deck slab supported by prestressed concrete or steel beams spaced at 2–3 m with typical depth ranges of 180 to 210 mm.

The slab specimen was made using concrete with the targeted C30/37 class according to Eurocode 2 [32]. The age of the tested slab was over 2 years; in order to determine real mechanical properties of concrete, three cubic specimens were prepared on the concreting day and were tested on the same day as slab testing. These specimens were cured at the same ambient temperature and humidity as the tested slab. The compressive strength of the concrete was determined based on the EN 12390-3 standard [33]. The average value of the compressive strength was 44 MPa.

The concrete slab was reinforced with 10 mm diameter solid GFRP (glass fiber reinforced polymer) bars with spiral ribbing. The mechanical properties of the GFRP bars were determined on five specimens using the ISO 10406-1 standard [34]. The average values of the tensile strength and modulus of elasticity of the GFRP rebars were 1000 MPa and 55 GPa, respectively.

As top and bottom reinforcement, bidirectional grids made of 10 mm diameter GFRP bars were used. The spacing of bars in both longitudinal and transverse directions was the same and equaled 80 mm. There were total of 11 longitudinal bars in the slab’s cross-section, and the concrete covers were 30 mm.

The slab specimen was constructed in a local precast concrete plant and transported to the laboratory. The slab was inspected before testing, and there were two initial cracks with widths less than 0.1 mm (due to handling and transportation or shrinkage), which were considered irrelevant.

The examination plan assumed loading and unloading the sample. Eight cycles were defined with different load rates. Loading was controlled by displacement. In the first three cycles, the increase was equal to 0.5 mm/min, and in the next five cycles, it was 2 mm/min. The following load values were achieved in subsequent cycles: 10 kN, 20 kN, 30 kN, 50 kN, 80 kN, 100 kN, 135 kN, and 150 kN. After the planned cycles, the slab was destroyed, but the examination presented here does not consider this stage.

**Figure 2:**
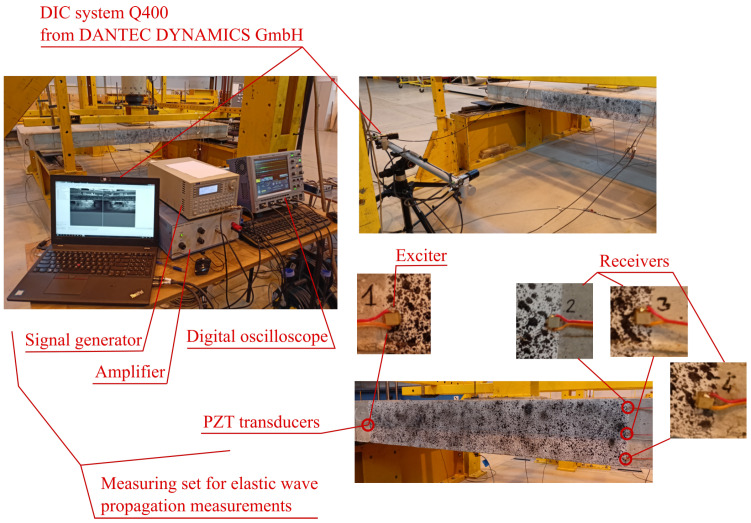
Measuring stand.

During the loading increase, the changes to the shape of the observed area were registered by the DIC system Q-400 by Dantec Dynamics GmbH (Ulm, Germany). For this purpose, the area was painted with irregular black speckles, as shown in Figure 2. In accordance with the rules for 3D DIC measurement [17], two high-resolution cameras (12 Mpx) were used. The distance between the cameras and the specimen was 150 cm, and the distance between the two cameras was 82 cm. The usage of DIC enabled not only the observation of crack propagation during loading but also the estimation of the level of permanent deformation after the load was removed. At the moment of crack appearance, part of the material is chipped, so the pattern in the neighborhood of the crack may be lost. Then, precise measurement of the crack opening is not possible. Therefore, to verify the experiment, traditional measurement of crack propagation was used. However, this measurement was performed on the opposite side of the slab.

For elastic wave propagation measurement, a set of four of the same PZT transducers (CMAP4 by Noliac, Kvistgård, Denmark) were glued onto the boundary of the observed area. The location of these sensors was selected to enable measurement of the wave in zones with different stress states. The wave was generated by an exciter on the left side in the middle of the height of the slab. The other three PZT transducers received the wave. The set for elastic wave measurement (see Figure 2) consisted of: signal generator, amplifier, four PZT sensors, preamplifier, and digital oscilloscope.

**Figure 3:**
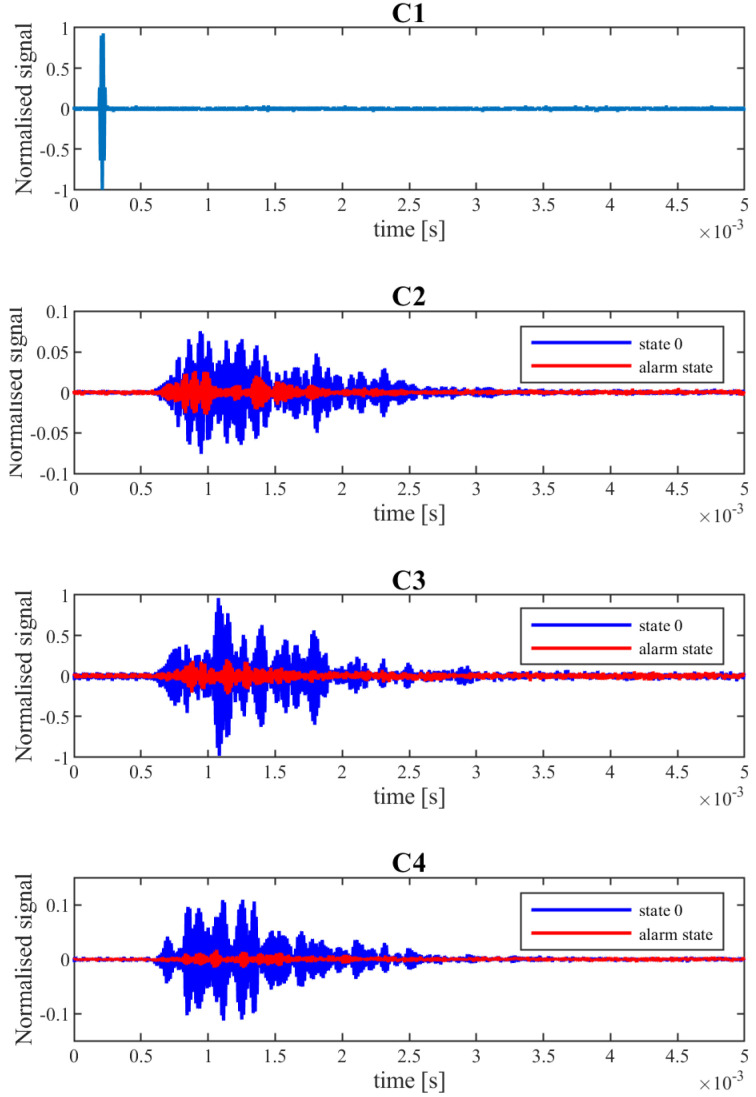
Examples of registered signals. The alarm state corresponds to a crack width of 0.7 mm.

The elastic wave measurements were made in states both without loading and under loading with the final value in each loading cycle. The generated signal was 3.5 sine waves, modified by a Hanning window, with an amplitude of 9 Vpp, and amplified 20 times. The operational frequency was 70 kHz. The normalized excitation signal is shown in Figure 3 C1. The signals excited/received by the PZT were named by the letter “C” and the number of the PZT sensor. Each recorded signal was averaged from 100 subsequent measurements. Finally, the set of registered patterns consisted of 23 element (8 measurements under loading and 15 with no force).

## 3. Results and Discussion

During the experiment, the signals registered by the receivers changed their amplitude and shape. Observed changes were related to the force applied to the slab and the appearance of cracks. To enable signal comparison, all signals were normalized using the biggest value of amplitude registered in all measurements (observed in the C2 signal). The normalized signals for the alarm state are shown in Figure 3 C2–C4 in comparison to State 0 (without loading and cracks and before the first loading cycle).

When GFRP reinforcement bars are used, crack limitations related to corrosion are not required due to the bars; corrosion-resistant properties, so larger crack widths can be tolerated compared to those for steel-reinforced concrete elements [35]. Standards [36,37,38] recommend a crack width limit of 0.5 mm to 0.7 mm. In this research, we defined an alarm state as the condition when the crack width reaches 0.7 mm.

**Figure 4:**
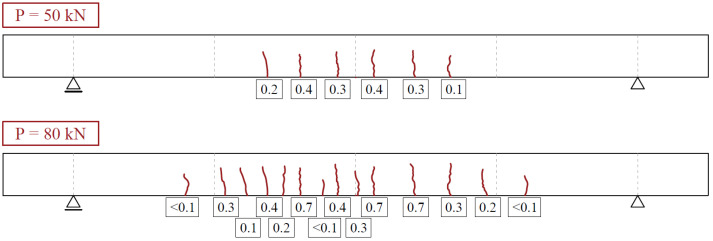
Crack patterns of tested slab at 50 and 80 kN load levels, respectively.

The first crack in the tested slab, as measured with the traditional way, appeared between the 30 kN and 50 kN load step at about 35 kN. At each load step, formation of cracks was evaluated, and crack widths were recorded and measured. The evolution of the crack pattern observed in the tests is shown in Figure 4 for two load levels: 50 kN and 80 kN, respectively. Only flexural cracks (no shear cracks) developed at both load levels.

**Figure 5:**
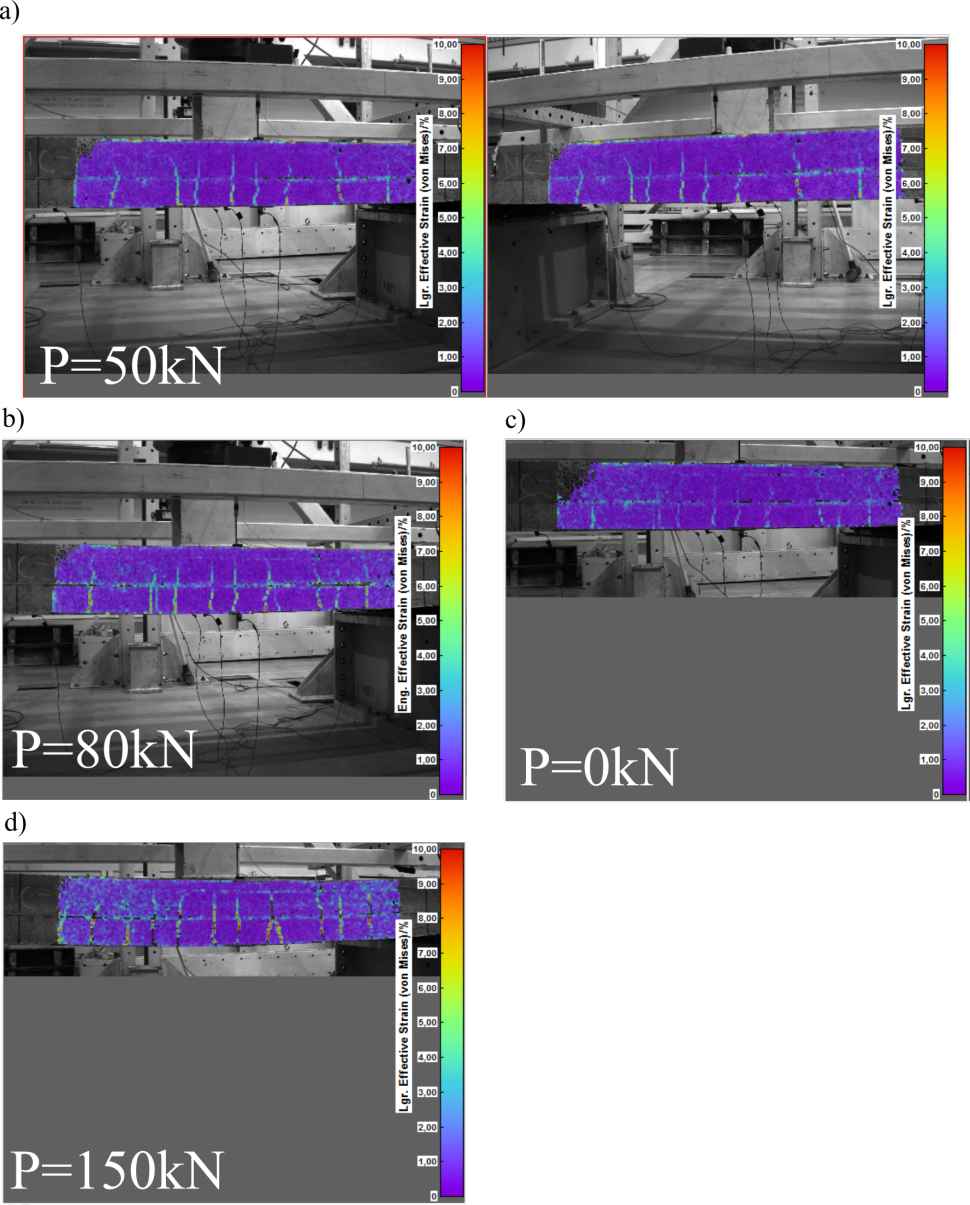
Lagrange effective strain distribution obtained with DIC application under different loading levels of the slab. In all figures, the same color scale is used (0–10%); (**a**) pictures registered by both cameras; (**b**–**d**) picture registered by the left camera.

The exemplary views registered by two cameras are shown in Figure 5a. Figure 5b–d show the pictures registered by the left camera; however, for obtaining the presented maps of Lagrange effective strain fields (according to the von Mises formula), photos made with both cameras were used. All DIC calculations were made in the ISTRA 4D commercial software. In Figure 5a,b,d, the strain fields were calculated with the first picture registered in a suitable loading cycle as a reference picture. Thus, in these figures, the increase in strain is visible with respect to permanent deformation remaining from the previous loading cycles. The permanent strain field after loading with 80 kN is shown in Figure 5c with the reference picture related to State 0 (before any load). The average accuracy of strain measurement for the analyzed region was 0.02%.

**Figure 6:**
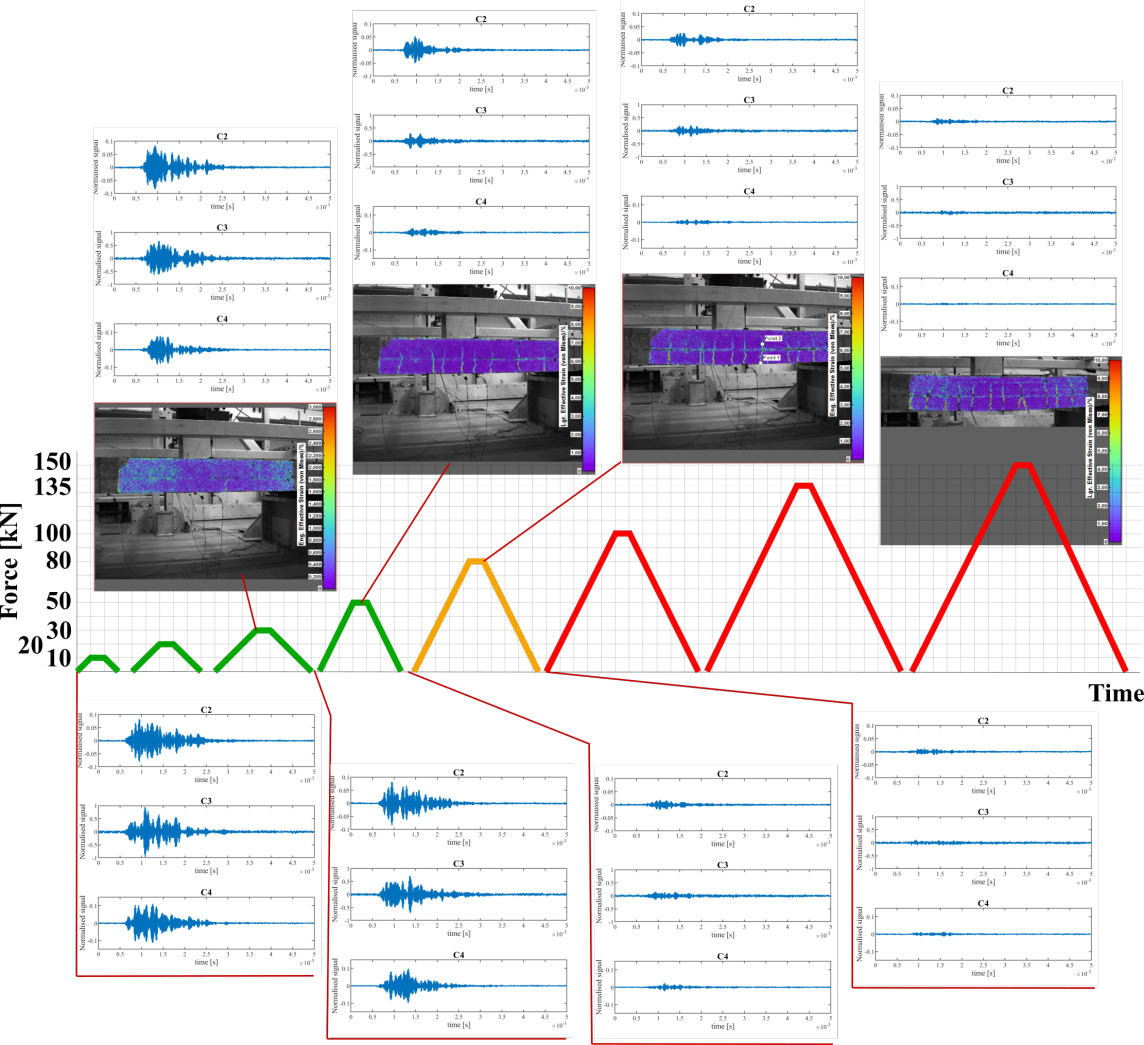
Loading cycles and their corresponding crack appearance and changes in the signals registered by the PZT sensors. Green indicates the loading cycles were signed and the slab was in a ‘healthy’ state. Orange indicates alarm loading cycles. The final cycles are marked with red and indicate the slab should be treated as destroyed.

The changes in the structure of the slab and the signals that appeared in selected loading cycles are summarized in Figure 6. The first significant crack was registered with DIC under the load P = 30 kN (before it was noticeable). Changes in the shape were observed in all registered signals. Especially, the amplitude of C4 (PZT sensor near the lower edge of the slab) decreased most significantly. However, after the load was relieved, the crack partially closed, which resulted in increasing the signal amplitude to the level from the beginning of the experiment, leaving changes only in the shape of the signal.

The P = 50 kN loading resulted in development of the first crack propagation as well as the appearance of new cracks. The changes in the structure were so big that all sensors registered a significant decrease in signal amplitude. This time, even after removal of the loading, the amplitudes did not reach the State 0 level. However, according to the previously mentioned rules for this type of structure, the slab could still be treated as undamaged.

**Figure 7:**
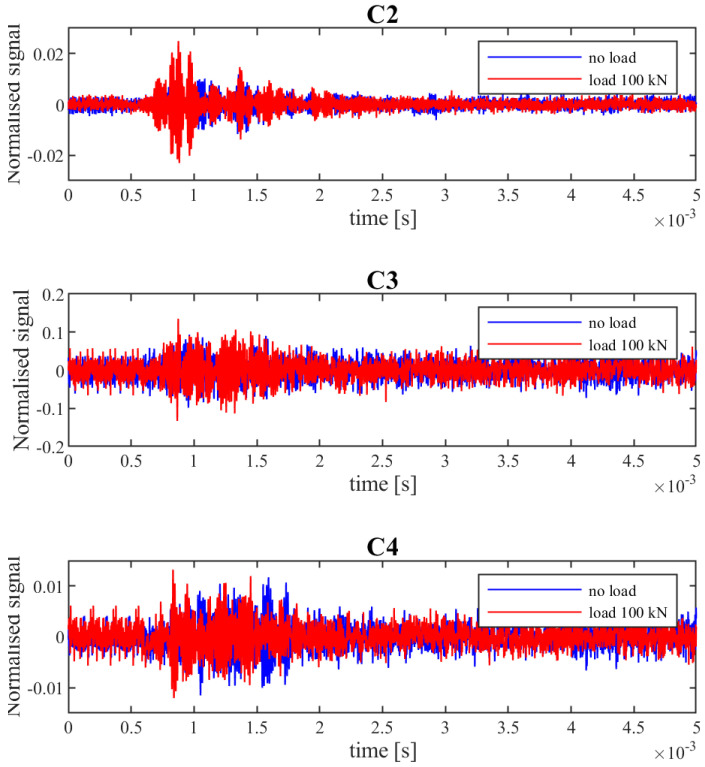
Examples of registered signals after the alarm state and with and without loading.

The damage caused by the P = 80 kN load were determined, based on standard measurement of crack openings, as needing an alarm. It should be mentioned that the amplitude of signals C2 and C3 when the structure was under the influence of force were bigger than prior to loading (P = 0 kN). This relationship was also observed in the next loading cycles, which hindered assessment of the condition of the structure. Examples of registered signals after the alarm state and with and without loading are shown in Figure 7. Easy designation of the level of signal amplitude below which a decrease would result in information about alarm/damage occurance was impossible.

To summarize, we must determine the influence that the amount and range of cracks and strain/stress state in the examined object or its parts has on elastic wave propagation. The changes in registered signals (considering amplitude and shape) have to be analyzed in connection with the location of the sensor.

Due to the inability of simple determination of parameters to enable structural health monitoring of the slab, an ANN was used. A shallow ANN with one hidden layer was selected. The architecture of this type of ANN can be symbolically described as X-H-O (X—the number of inputs collected in input vector **X**, H—the number of hidden neurons, and O—the number of ANN outputs collected in vector **O**).

**Figure 8:**
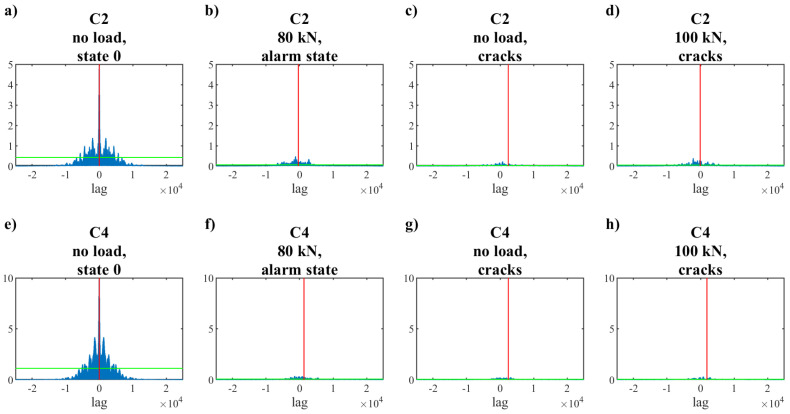
Examples of absolute values from correlation of the signals. The base signal for each selected sensor was its normalized signal from State 0. The centers of gravity of the presented functions were calculated and are shown on the crossing of red and green lines.

Regarding the length of the registered signals and the number of collected patterns, it was necessary to reduce the number of parameters describing each signal by selecting the most significant of them. With regard to the dependencies presented above, cross-correlation of the signals was calculated. As a base signal for each selected sensor, the normalized signal from State 0 was used, and the signals from other measurements within a given sensor were compared to it. Then, the absolute value of the cross-correlation vector was calculated. Some results are shown in the diagrams in Figure 8a–d (for C2—signal from PZT near the top edge) and in Figure 8e–h (for C4). In Figure 8c,g, the correlation for the signal registered after the alarm loading level is presented (without loading, P = 0). The centers of gravity of considered functions were calculated, and then this information was used as input parameters for ANN.

Two cases of ANN were considered. The difference lay in the number of input parameters, as shown below:

- Case 1: **X** = {xC2; yC2; xC4; yC4},
- Case 2: **X** = {xC2; yC2; xC4; yC4; Load},

where (xC2, yC2)—the coordinates of the center of gravity of the absolute value of the cross-correlation vector of signal C2, (xC4, yC4)—the same as above but for the C4 signal, and load—the information about loading: Load = 0 means that there is no load on the structure; Load = 1 means that some load is applied.

In both analyzed cases, the output vector **O** = {$O_{1}$} consisted of one element, which was the class number. $O_{1}$ = 0 means that the structure is without damage, and $O_{1}$ = 1 suggests an alarm condition (the structure requires inspection because of the cracks).

All 23 patterns collected during the measurement were divided into two sets: learning and testing. The learning set included the patterns corresponding to P ∈ {10, 20, 30, 80, 100, 135, and 150} kN and eight patterns for P = 0 kN but with different levels of crack growth. The testing set consisted of seven patterns connecting to P = 0 kN (different crack levels) and one specimen under loading with P = 50 kN. During learning procedures, the ANN did not use the testing patterns. The testing procedure was extended to two steps. In the first, testing using the above-mentioned testing patterns was made. In the second step, based on patterns registered during measurement, a new set of signals was created by the addition of random noise to the original patterns. Using this set enabled testing of the specimen loaded by force with a different magnitude (in the primary testing set, only one pattern was under loading).

**Figure 9:**
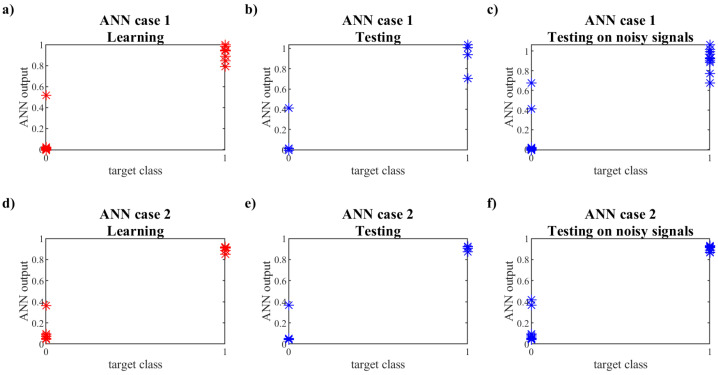
ANN results.

The ANNs were trained and testing according to the Levenberg–Marquardt algorithm using the approach described in [26]. The determination of the slab condition (class of the structure) was made by a set of ANNs, not a single network. Due to the small group of learning patterns, the number of hidden neurons was limited in Case 1 to three and in Case 2 to two neurons. In Figure 9, the results of learning, testing, and testing with noisy signals are shown for each case separately. Each star in these diagrams represents the classification of the slab condition made by the set of ANNs for a single pattern. If the target class is 0 (no damage), the output should be in the range of 0 to 0.5, and if the target class is 1, the output should be between 0.5 and 1. The more the network outputs are concentrated around the value describing the class, the better. As can be seen in Figure 9, the results of Case 2, in which the information about loading is included as an input parameter, are better. It enables the proper classification of the specimen’s condition for every pattern.

## 4. Conclusions

The elastic wave propagation phenomenon can be useful in the determination of the state of concrete elements under bending. Considering the discontinuities in materials caused by cracks and the influence of strain fields on wave propagation, the information collected in both tensile and compressive zones has to be included simultaneously. The proposed method allows determining the state of a slab when the initial state of the structure is “healthy”, which means a lack of significant cracks at the beginning of monitoring.

It has to be emphasized that the signals generated and registered by PZT transducers are sensitive to the way they are glued; concrete is an inhomogeneous material, and during manufacturing of reinforced elements, inaccuracies may appear. The above-mentioned factors, as well as the type of applied reinforcement or concrete, influence wave propagation. In our opinion, the development of a universal tool for monitoring the structural health of concrete elements will be a difficult or perhaps even impossible task. However, after narrowing down the set of variables and widening the collection of patterns (for statistical consideration), application of artificial intelligence automation of damage detection in concrete elements seems to be real.

## Data Availability

Research data will be made available upon written request of the interested party.
